# 'How Do I Cure a Ghost?' From Story-Telling to Sense-Making: Exploring Psychiatrist Perspectives of Cultural Competence

**DOI:** 10.1192/bjo.2025.10257

**Published:** 2025-06-20

**Authors:** Nawal Benachar

**Affiliations:** North London NHS Foundation Trust, London, United Kingdom. University of Oxford, Oxford, United Kingdom

## Abstract

**Aims:** The culture of psychiatry in the UK is deeply rooted in Western biomedical paradigms, raising questions around its ability to meet the needs of culturally diverse patients. Literature consistently demonstrates that individuals from diverse backgrounds experience disproportionately poorer mental health outcomes, highlighting the need for a more culturally responsive approach to care.

This research examines how the concept of 'cultural competence’ is understood by current trainee and consultant psychiatrists, through the retelling of their experiences of navigating culture in the clinic.

This research aimed to (1) explore perspectives of UK psychiatrists of navigating culture in the clinic, and to (2) evaluate the effectiveness of the current postgraduate psychiatric curriculum in fostering cultural competence.

**Methods:** Employing a narrative inquiry approach, the primary source of data was collected through open interviewing to promote the process of storytelling. Three clinicians of various clinical grades were recruited, and five narratives extracted for analysis. Data was analysed using Labov’s method of structural organisation.

**Results:** The findings demonstrate that clinicians consistently frame their encounters with cultural diversity as challenging, often conceptualising them as conflicts to be overcome. Three core competencies for effective cross-cultural practice emerged:

1. *Curiosity and openness.*
2. *Polycultural practice.*
3. *Critical evaluation of resources.*

These competencies informed the development of a new proposed model of cultural competence designed to guide educators in fostering these qualities in psychiatric trainees.

**Conclusion:** The findings highlight a significant gap in the existing postgraduate psychiatric curriculum, suggesting that current medical education frameworks are insufficiently aligned with the demands of a multicultural society. The study advocates for comprehensive curricular reform that starts with a shift in the underlying conceptual frameworks of psychiatric education, encouraging practitioners to adopt a more holistic, culturally sensitive approach to mental health care. Only once this conceptual foundation is established can practical reforms effectively follow, ensuring that trainees develop not only technical competence but also the cultural insight necessary for inclusive, empathetic care.

